# Pyrazole and Triazole Derivatives as *Mycobacterium tuberculosis* UDP-Galactopyranose Inhibitors

**DOI:** 10.3390/ph15020197

**Published:** 2022-02-04

**Authors:** Dalia M. Ahmed, Jeffrey M. Chen, David A. R. Sanders

**Affiliations:** 1 Department of Chemistry, University of Saskatchewan, 110 Science Place, Saskatoon, SK S7N 5C9, Canada; dalia.ahmed@usask.ca; 2 Pharmaceutical Chemistry Department, Faculty of Pharmacy, Ain Shams University, Cairo 11566, Egypt; 3 Vaccine and Infectious Disease Organization, Saskatoon, SK S7N 5E3, Canada; jeffrey.chen@usask.ca; 4 Department of Veterinary Microbiology, Western College of Veterinary Medicine, University of Saskatchewan, Saskatoon, SK S7N 5B4, Canada; 5 Vaccinology and Immunotherapeutics Program, School of Public Health, University of Saskatchewan, Saskatoon, SK S7N 2Z4, Canada

**Keywords:** pyrazoles, triazoles, UDP-galactopyranose mutase, inhibitors, antituberculosis, enzyme kinetics

## Abstract

UDP-galactopyranose mutase (UGM) is an essential enzyme involved in the bacterial cell wall synthesis, and is not present in mammalian cells. Thus, UGM from *Mycobacterium tuberculosis* (*Mtb*) represents a novel and attractive drug target for developing antituberculosis agents. A pyrazole-based compound, **MS208**, was previously identified as a mixed inhibitor of *Mtb*UGM which targets an allosteric site. To understand more about the structure activity relationship around the **MS208** scaffold as a *Mtb*UGM inhibitor, thirteen pyrazoles and triazole analogues were synthesized and tested against both *Mtb*UGM and *Mycobacterium tuberculosis* in vitro. While the introduced structural modifications to **MS208** did not improve the antituberculosis activity, most of the compounds showed *Mtb*UGM inhibitory activity. Interestingly, the pyrazole derivative **DA10** showed a competitive model for *Mtb*UGM inhibition with improved Ki value of 51 ± 4 µM. However, the same compound did not inhibit the growth of *Mycobacterium tuberculosis*.

## 1. Introduction

Tuberculosis (TB) is an infectious disease that remains one of the top ten causes of death worldwide [1]. *Mycobacterium tuberculosis* (*Mtb*), which is the causative agent of TB, has developed resistant strains against many of the clinically available antibacterial agents [1]. The crisis of multidrug-resistant *Mtb* has continued and there is now an increasing demand to develop new potential drug candidates that target novel biosynthetic pathways of *Mtb* [1].

**Figure 1:**
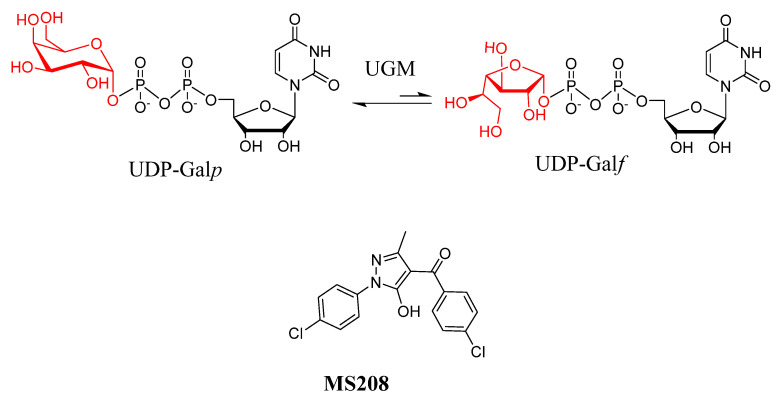
Reaction catalyzed by UGM by forming UDP-Gal*f* from UDP-Gal*p*, and the chemical structure of **MS208**.

UDP-galactopyranose mutase (UGM) is an essential flavoenzyme for the growth of *M. tuberculosis* as it catalyzes the interconversion between UDP-galactopyranose (UDP-Gal*p*) to UDP-galactofuranose (UDP-Gal*f*). The latter is one of the building blocks of the bacterial cell wall (Figure 1) [2,3]. Thus, *Mtb*UGM represents a novel potential bacterial target. Gal*f* is not found in mammals, and therefore there is a greater selectivity for the inhibition of this synthetic pathway [4].

Inhibitors of UGM can be classified according to their resemblance to the natural substrate as either substrate-like inhibitors or non-substrate-like inhibitors [5,6,7,8]. **MS208** is a pyrazole based molecule which was recently identified as a non-substrate-like inhibitor of *Mtb*UGM (Figure 1) [4]. Saturation Transfer Difference NMR (STD-NMR) spectroscopy studies showed that **MS208** competes indirectly with UDP-Gal*p* [9]. Molecular modeling studies suggested that **MS208** binds to an allosteric site, a distal site from the active site [9]. Kinetic inhibition studies revealed that **MS208** is a mixed inhibitor of *Mtb*UGM [9]. A mixed inhibitor means that the inhibitor molecule binds to and inhibits both the enzyme and the enzyme-substrate complex. Site-directed mutagenesis studies supported the hypothesis of **MS208** being an allosteric site inhibitor as two allosteric site mutants, Y253A and D322A, were not inhibited by **MS208** [9].

Although previous studies involved testing of pyrazolo analogues as antituberculosis molecules, they were not tested against *Mtb*UGM except for **MS208** [10,11,12]. No information is available about the structure activity relationship (SAR) around the **MS208** scaffold as *Mtb*UGM inhibitor. Also, many triazolo derivatives were reported to have antituberculosis activity but none have been tested against *Mtb*UGM as a potential molecular target [13,14].

In this work we synthesized novel compounds of pyrazole and triazole nuclei and tested their activities in vitro against *Mtb* and *Mtb*UGM.

## 2. Results and Discussions

**Figure 2:**
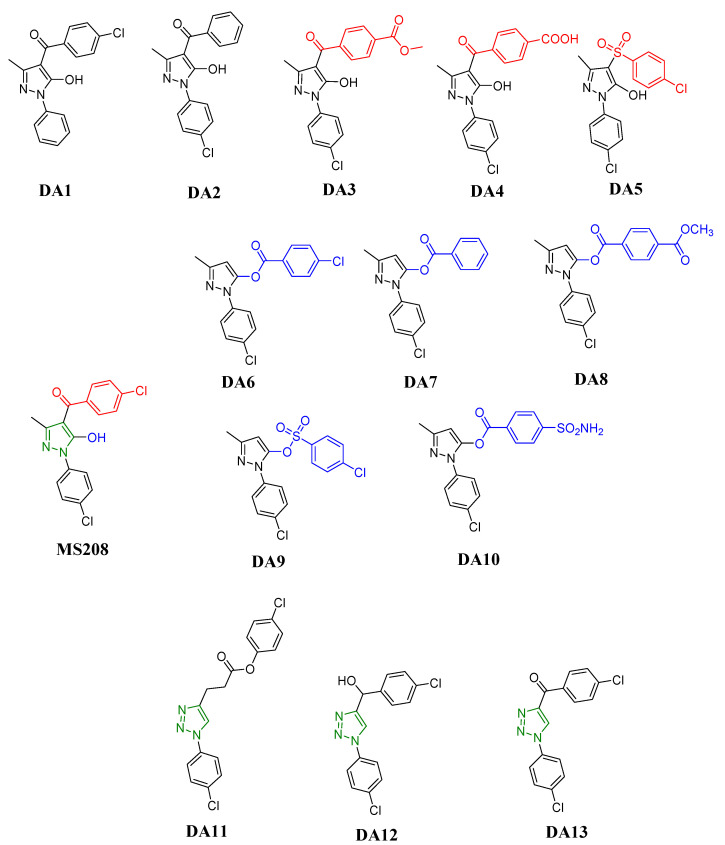
Chemical structures of targeted compounds **DA1**–**DA13**. *C*-acylated pyrazole series included **DA1**–**DA5**, *O*-acylated pyrazole series included **DA6**–**DA10** and triazolo series **DA11**–**DA13**.

The reported computational binding model of **MS208** proposed that the main interactions between **MS208** and the allosteric site were mainly hydrophobic interactions [9]. To explore the role of the halogen in binding, a chlorine atom from either of the rings was deleted in compounds **DA1** and **DA2** (Figure 2). Also, the same model suggested that the introduction of polar groups to either of **MS208** phenyl rings would increase the binding via polar interactions with the polar residues located at the periphery of the allosteric site [9]. Accordingly, to test this proposed hypothesis, polar groups were introduced at the para position of the benzoyl part (namely an ester group in **DA3** and a carboxylate in **DA4** (Figure 2). Lastly, since a sulfonyl group had two hydrogen bond acceptors, we substituted the carbonyl group in **MS208** with a sulfonyl in **DA5** (Figure 2), looking for additional hydrogen bond interactions with the nearby allosteric site residues. To investigate the role of the 5-OH group as an hydrogen bond donor feature, we masked this OH in *O*-acyl series which included **DA6**–**DA10** (Figure 2).

Three novel analogues having a triazolo core, **DA11**–**DA13** (Figure 2), were designed to explore the effect of the presence of a third nitrogen as a hydrogen bond acceptor in the binding.

### 2.1. Synthesis of the Target Compounds

**Scheme 1:**
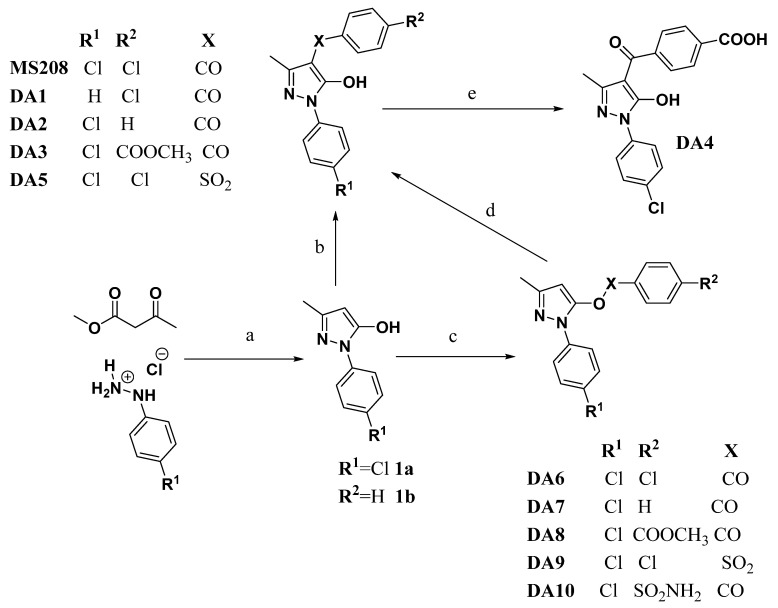
Pyrazolo analogues synthesis. Reactants and conditions: (**a**) absolute EtOH, reflux 3 h, (**b**) dry Ca(OH)_2_, anhydrous 1,4-dioxane 80 °C, RCOCl (1.1 equivalent), 3M HCl, (**c**) TEA, chloroform, 0 °C then RCOCl, reflux, (**d**) **DA8**, anhydrous K_2_CO_3_, anhydrous 1,4-dioxane, reflux, 1M HCl, (**e**) **DA3**, K_2_CO_3_, methanol, water, reflux.

Synthesis of pyrazole derivatives **MS208** and **DA1**–**DA10** followed the synthetic route described in Scheme 1. Generally, the 1-aryl-3-methyl-5-pyrazolone (**1**) is synthesized via condensation between arylhydrazine and acetoacetic acid esters in a polar solvent [15,16]. Since C4 behaves as active methylene due to keto-enol tautomerism, the hydrogen of that active methylene can undergo acylation reaction [17]. Selectivity of the acylation reaction, either *C*-acylation at C4 or *O*-acylation of the OH, is controlled by the choice of the base used and the solvent in which the reaction happens [18,19,20]. For *O*-acylation, triethylamine was the base and chloroform was the solvent while for *C*-acylation calcium hydroxide was the base and anhydrous 1,4-dioxane was the solvent [18,20]. The strong base, calcium hydroxide, was needed to form the enolate anion [20]. Also, the calcium cation complexed the enolate anion oxygen to prevent the nucleophilic attack to the carbonyl carbon of the acid chloride, thus preventing the formation of the *O*-acylated product [20].

*C*-acylation was confirmed by ^1^H-NMR by monitoring the disappearance of the C4 proton signal of the starting material. Moreover, the appearance of two new doublets, each integrating to two protons of the *p*-chlorobenzoyl, *p*-chlorobenzosulfonyl and in **MS208**, **DA1** and **DA5**, respectively, was indicative of successful introduction of these groups.Similarly, the appearance of multiplet signal integrating to five protons, in the case of compound **DA2** confirmed the formation of the expected product.

Compound **DA3** could not be obtained by direct *C*-acylation but was obtained from the *O*-acylated isomer using anhydrous potassium carbonate via the Fries rearrangement mechanism [21]. Alkaline hydrolysis of the ester group of compound **DA3** yielded the corresponding carboxylic acid derivative **DA4** following a reported method in the literature [22]. Successful ester hydrolysis was verified by the disappearance of the singlet signal, integrating to three protons upfield, corresponding to the methyl group of the ester of compound **DA3**. Additionally, a broad singlet signal downfield of the carboxylate proton was characteristic of **DA4** structure.

**Scheme 2:**
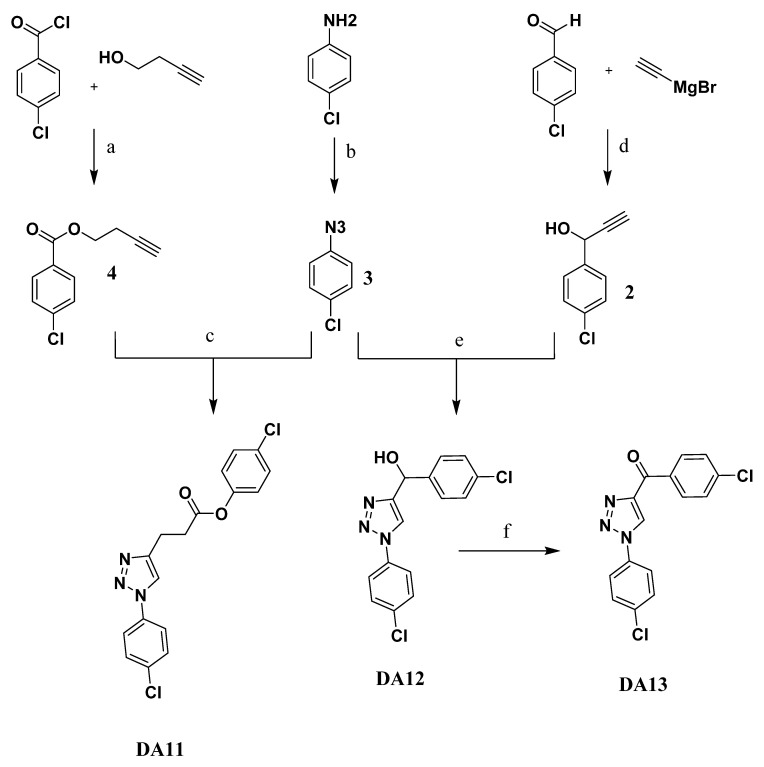
Triazolo analogues synthesis. Reactants and conditions: (**a**) CH_2_Cl_2_, TEA, RT, (**b**) NaNO_2_/conc. HCl, H_2_O then NaN_3_, (**c**) CH_2_Cl_2_, CuSO_4_·H_2_O, Na ascorbate, RT, (**d**) anhydrous THF(**e**) t-BuOH:H_2_0 1:1, CuSO_4_·H_2_O, Na ascorbate, RT, (**f**) CH_2_Cl_2_, MnO_2_, RT.

Synthesis of **DA12**–**DA13** followed Scheme 2. Generally, to prepare 1,2,3-triazolo derivatives, a cycloaddition between azide derivatives and alkyne derivatives is catalyzed by CuI [23]. The common starting material for both pathways, azido *p*-chlorobenzene, was obtained from *p*-chloro aniline via diazotization, and the product entered the second step without any further purification [24]. *p*-Chloro benzaldehyde was reacted with the Grignard reagent, ethynyl magnesium bromide, to produce **2** [25]. Esterification of *p*-chlorobenzoyl chloride using 3-butyn-1-ol in the presence of TEA yielded compound **4** [26]. Both alkynyl compounds entered the second step as is.

Azide-alkyne cycloaddition of **4** with **3** easily gave **DA11**. Copper-catalyzed cyclo-addition of **2** with azido *p*-chlorobenzene gave compound **DA12**. Oxidation of **DA12** by MnO_2_ afforded **DA13** following a reported procedure [25].

^1^H-NMR of **DA11** showed two triplet aliphatic signals at δ 3.21 and 4.58 ppm and four doublet aromatic signals for the two aromatic *p*-substituted rings from δ 7.60 to 7.95 ppm. The aromatic hydrogen of the triazolo ring appeared downfield at δ 8.77 ppm as a singlet signal. ^1^H-NMR proved the formation of **DA12** by the presence of four aromatic doublet signals for the two aromatic *p*-substituted rings from δ 7.39 to 7.94 ppm. The hydrogen of the triazole ring appeared downfield at δ 6.24 ppm as a doublet signal due to coupling with the benzylic proton which appeared as doublet signals at δ 5.90 ppm. Oxidation of **DA12** to obtain **DA13** was successful as shown by the disappearance of the benzylic proton signal in the ^1^H-NMR and appearance of carbonyl carbon at δ 184.0 ppm in ^13^C-HMR spectra.

### 2.2. Antituberculosis Activity

To evaluate the activity of the synthesized compounds against *M. tuberculosis*, minimum inhibitory concentration (MIC) values were determined using the Resazurin Microplate Assay (REMA) method (Table 1) [27,28]. The REMA principle relies on using a blue non-fluorescent dye (resazurin), that is reduced by viable, metabolic active bacteria to a pink fluorescent (resorufin). Resorufin is easily visualized and quantified by fluorescence (excitation and emission wavelengths of 530 and 590 nm, respectively) [27]. REMA is superior to the Colony Forming Unit (CFU) method, which relies on enumerating bacteria that require several weeks of growth on agar-based solid culture media, as REMA provides results in days instead of weeks, and requires smaller amounts of test compounds [29]. The REMA MIC value of **MS208** was 12.5 µg/mL, which was comparable to values reported in the literature [10]. The absence of any of the chlorine atoms in **MS208** phenyl groups did not affect MIC, as shown by the same MIC value for **DA1** and **DA2**.

**DA3** and **DA4** showed higher MIC values compared to **MS208,** which implied that the introduction of polar groups in the para position did not improve the antituberculosis activity of these derivatives. **DA4** showed less antituberculosis activity compared to the ester analogue **DA3**, presumably due to the ionized nature of the carboxylic group in **DA4** preventing the molecule from crossing the lipophilic mycobacterium cell wall.

The acyl derivative, **MS208,** is more active than the sulfonyl derivative, **DA5**. All *O*-acyl analogues (**DA6**–**DA10**) showed less activity compared to **MS208**, indicating that the presence of the unmasked OH group was important for the antituberculosis activity of the pyrazolo analogues. Three triazolo analogues (**DA11**–**DA13)** were less potent than **MS208** as well, which suggested that the pyrazolo analogues were superior to the triazolo derivatives.

### 2.3. MtbUGM Inhibitory Activities

#### 2.3.1. Kinetic Inhibition

**Figure 3:**
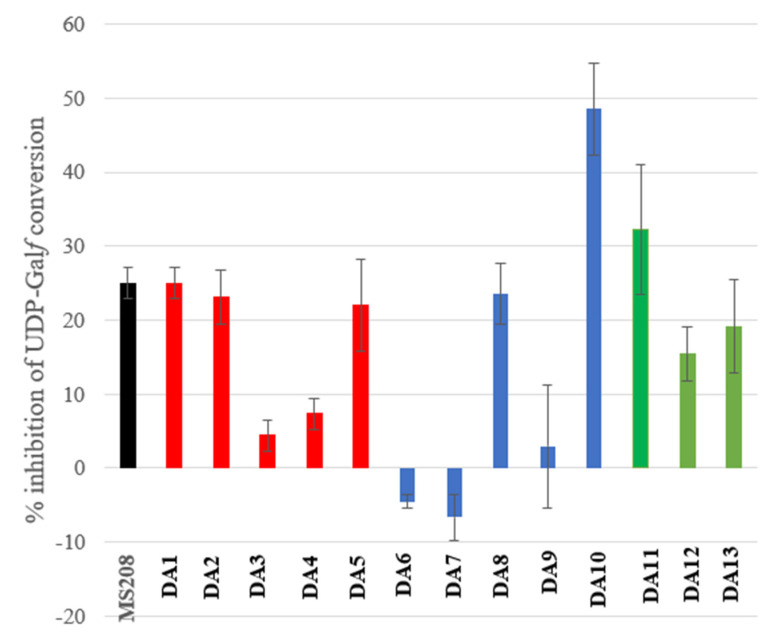
% Inhibition of *Mtb*UGM at 15 µM of UDP-Gal*f* using 60 µM of the test compounds. % Inhibition bar of **MS208** in black, *C*-acyl pyrazole derivatives (**DA1**–**DA5**) in red, *O*-acyl pyrazole derivatives (**DA6**–**DA10**) in blue and triazolo derivatives (**DA11**–**DA13**) in green.

Determination of the % inhibition of *Mtb*UGM activity by the synthesized compounds followed a reported HPLC procedure. In this method, the conversion of UDP-Gal*f*, to UDP-Gal*p* (reverse reaction) was carried out. The reaction was quenched using n-butanol at a time point that gave 30–40% conversion. The substrate and product were then separated and quantified by HPLC. The results of % inhibition of *Mtb*UGM by 60 µM of the synthesized compounds using 15 µM UDP-Gal*f* are shown in (Figure 3).

**DA1** and **DA2** showed comparable inhibition to that of **MS208**, indicating the removal of one of the chlorine atoms on either of the phenyl rings did not affect inhibition, which was consistent with the antituberculosis activity results. Contrary to the proposed hypothesis, the introduced polar substituents did not improve *Mtb*UGM inhibition as shown by the lower % inhibition values of **DA3** and **DA4** compared to that of **MS208**. The sulfonyl derivative, **DA5**, showed comparable % inhibition to that of the carbonyl derivative **MS208**. Three of the *O*-acyl analogues, **DA6**, **DA7** and **DA9**, did not show inhibition of *Mtb*UGM, however; two of these *O*-acyl analogues, **DA8** and **DA10** still showed comparable % inhibition to **MS208.** The different inhibition pattern between *O*-acyl analogues, suggested that they are binding differently to *Mtb*UGM. The triazolo analogues, **DA11**-**DA13**, showed comparable % inhibition to that of **MS208**.

#### 2.3.2. Kinetic Inhibition Mechanism of **DA10**

Since **DA10** was the most promising inhibitor, showing 50 % inhibition of *Mtb*UGM activity at 60 µM of the test compound, characterization of the kinetic inhibition model of **DA10** was followed.

**Figure 4:**
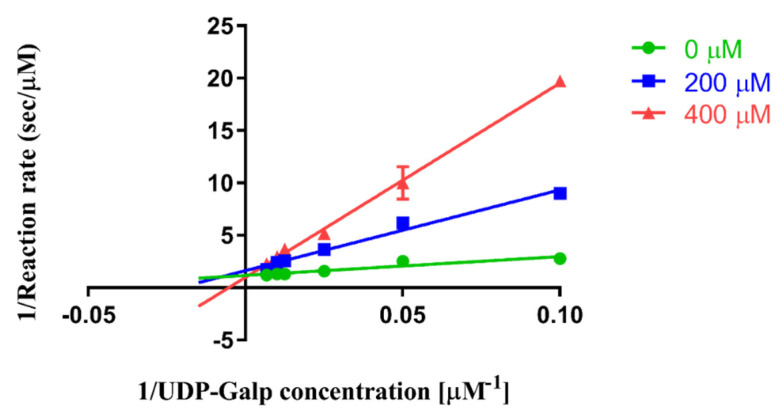
Lineweaver Burk plot of *Mtb*UGM activity in presence of **DA10** at three different concentrations (0, 200, and 400 µM).

The same HPLC-based kinetic assay was followed while varying concentrations of **DA10** with changing concentrations of UDP-Gal*f*. A Lineweaver Burk plot showed intersecting lines on the *y*-axis (Figure 4).

**Figure 5:**
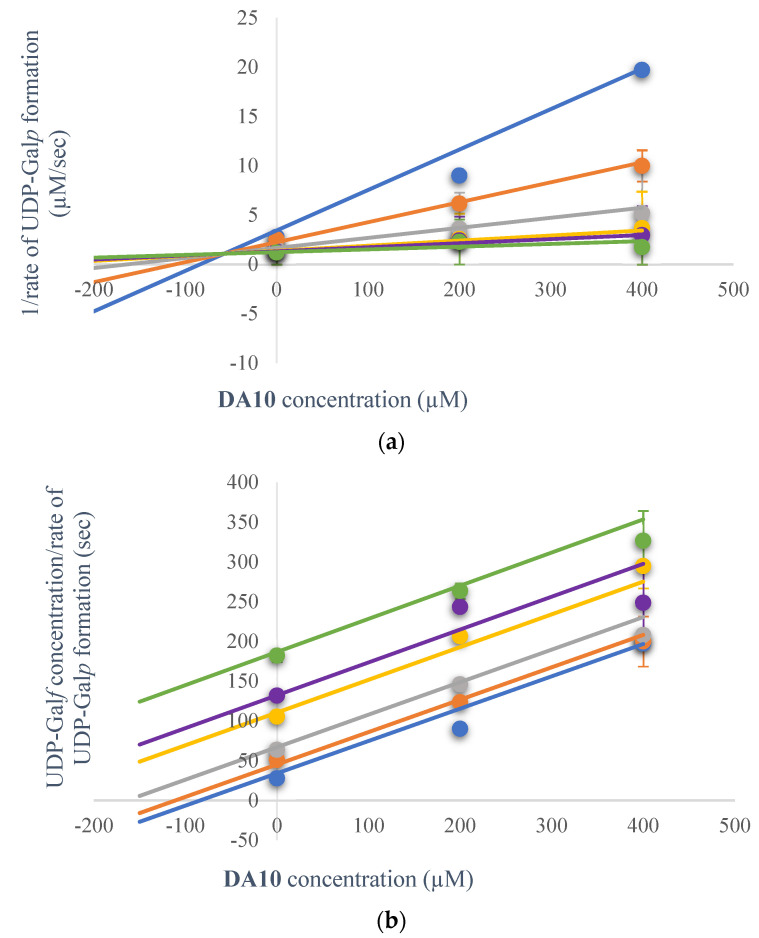
Diagnostic plots areshowing competitive model of inhibition of *Mtb*UGM by **DA10**: (**a**) Dixon plot has crossing lines in the fourth quadrant; (**b**) Cornish-Bowden plot is showing parallel lines.

In the Dixon plot, the lines intersected above the *x*-axis in the fourth quadrant, while in the Cornish-Bowden plot the lines were parallel. These plot patterns are characteristic of competitive inhibition (Figure 5).

Although **MS208** is a mixed inhibitor, the analogue **DA10** is a competitive inhibitor. **DA10** prevents the substrate from binding to the enzyme, while **MS208** prevents the substrate from binding to the enzyme as well as prevents the enzyme-substrate complex from converting to product. A competitive inhibitor simply means the inhibitor binding is mutually exclusive with the substrate binding, but gives no information about the binding site. The Ki value generated from the global fitting by GraphPrism was 51 ± 4 µM.

## 3. Materials and Methods

### 3.1. Chemistry

Chemicals were obtained from commercial suppliers (Sigma-Aldrich (Oakville, ON, Canada), Alfa Aesar (Haverhill, MA, USA, and Fisher Scientific (Waltham, MA, USA) and used without further purification. Glassware was cleaned with organic solvents, mainly acetone, and dried in an oven. 1,4-Dioxane was dried prior to use by refluxing with sodium metal and testing anhydrous conditions using benzophenone. The dried 1,4-dioxane was stored under anhydrous condition using 3.0 Å molecular sieves under N_2(g)_. THF was obtained as a fresh distillate from a solvent purifier system, and stored with 3.0 Å molecular sieves under N_2(g)_. All anhydrous reactions were run under N_2(g)_.

To monitor the progress of the reactions, the reaction mixtures were spotted against the corresponding starting material on pre-coated TLC plates (Merck Kieselgel 60F254, 0.25 mm thickness) and eluted using the appropriate solvent system. Detection of the organic compounds on the TLC plates was carried out using UV light at 254 nm. Fisher Scientific Silica Gel 60 was used for flash chromatography (FCC) (230–400 mesh).

Structural elucidations of the final compounds included the following characterization experiments: ^1^H-NMR, ^13^C-NMR, (see Supplemantry Materials) and High-Resolution Mass Spectrometry (HRMS). A Bruker 500 MHz spectrometer was used to obtain the NMR spectra after dissolving the compound in the appropriate deuterated solvent (CDCl_3_ or DMSO-d_6_). Chemical shifts (δ) are reported in parts per million (ppm) units downfield relative to the deuterated solvent signal. HRMS was performed on a QSTAR XL MS/MS System. All of the structural analyses (NMR and HRMS) were performed in the Saskatchewan Structural Sciences Centre (SSSC), U of S.

#### General Procedure for the Syntheses of Starting Materials

Synthesis of the starting material, **1**–**4**, followed reported procedure [10,24,25,26,30].

**MS208, DA1 and DA5** synthesis:

The procedure for the synthesis of **MS208**, analogues **DA1** and **DA5** was adapted from a reported procedure [20]. The appropriate starting material **1a** or **1b** (1 molar equivalent) was gently heated in anhydrous 1,4-dioxane till fully dissolved. Anhydrous Ca(OH)_2_ (2 molar equivalent) was added and the reaction mixture stirred under at 80 °C for 30 min. The reaction was cooled down to room temperature and the appropriate acid chloride (1.1 molar equivalent) was added dropwise while stirring. The reaction mixture was further refluxed at 110 °C while stirring for an additional 3 h. Reaction progress was monitored using TLC (EtOAc:hexane 2:1). Upon reaction completion, solvent was removed under vacuum. The residue left was dissolved in smallest amount of EtOAc and 3M HCl (25 mL/0.5 mmol **1a** or **1b**) was added. The mixture was left stirred for 30 min followed by extraction of the crude product using 3X EtOAc. The organic layers were combined and washed with water, brine and dried over anhydrous Na_2_SO_4_. EtOAc was removed under a vacuum to obtain a product which was purified using either using FCC or recrystallization using the appropriate solvent.

4-*p*-chlorobenzoyl-1-*p*-chlorophenyl-3-methyl-5-pyrazol (**MS208**): Purified using recrystallization using absolute EtOH and 10% *v*/*v* 3M HCl, yield 45%, ^1^H-NMR (CDCl_3_, δ ppm): 2.10 (s, 3H), 7.42 (d, 2H, *J* = 9.0 Hz), 7.45 (d, 2H, *J* = 8.6 Hz), 7.58 (d, 2H, *J* = 8.6 Hz), 7.84 (d, 2H, *J* = 9.0 Hz), ^13^C-NMR (CDCl_3_, δ ppm): 15.9, 103.6, 121.7, 128.8, 129.3, 129.4, 132.2, 135.7, 135.8, 138.4, 147.9, 161.6, 190.4, HRMS (FD+) *m*/*z*: [M]+ Calcd for C_17_H_12_Cl_2_N_2_O_2_ 346.0276; Found 346.0265.

4-*p*-chlorobenzoyl-1-phenyl-3-methyl-5-pyrazol (**DA1**): Purified using FCC Hex:EtOAc 1:1, yield 45%, ^1^H-NMR (CDCl_3_, δ ppm): 2.12 (s, 3H), 7.32 (t, 1H, *J* = 7.3 Hz), 7.47 (m, 2H), 7.50 (d, 2H, *J* = 8.3 Hz), 7.60 (d, 2H, *J* = 8.3 Hz) and 7.86 (m, 2H), ^13^C-NMR (CDCl_3_, δ ppm): 15.8, 103.5, 120.8, 126.8, 128.8, 129.0, 129.1, 136.2, 138.0, 138.1, 147.3, 160.1, 190.3, HRMS (FD+) *m*/*z*: [M]^+^ Calcd for C_17_H_13_Cl_1_N_2_O_2_ 312.0665; Found 312.0672.

4-*p*-chlorobenzosulfonyl-1-*p*-chlorophenyl-3-methyl-5-pyrazol hydrochloride (**DA5**): Purified using FCC Hex:EtOAc 2:1, yield 15%, ^1^H-NMR (CDCl_3_, δ ppm): 2.23 (s, 3H), 7.31 (d, 2H, *J* = 8.8 Hz), 7.40 (d, 2H, *J* = 8.8 Hz), 7.54 (d, 2H, *J* = 8.8 Hz) and 7.70 (d, 2H, *J* = 8.8 Hz), ^13^C-NMR (CDCl_3_, δ ppm): 12.2, 124.2, 124.5, 127.5, 128.6, 128.9, 129.3, 129.6, 129.7, 129.8, 129.9, 133.0, 133.6, 137.7, 142.3, 147.1, HRMS (FD+) *m*/*z*: [M]+ Calcd for C_16_H_12_Cl_3_N_2_O_3_S 383.2430; Found 383.2481.

**DA2 and DA3 synthesis:**

Fries rearrangement of *O*-acyl compounds, **DA7** and **DA8**, to give *C*-acyl compounds, **DA2** and **DA3**, respectively, was carried out following an adapted method from literature [31]. 1 Molar equivalent of the appropriate *O*-acyl compound dissolved in anhydrous 1,4-dioxane then 2 molar equivalent of anhydrous K_2_CO_3_ was added. The reaction was stirred overnight at 90 °C under N_2(g)_. Solvent evaporated under vacuum and the residue dissolved in minimum amount of EtOAc. 3M HCl was added and stirred with the product for 1 h. The organic layer was collected, and the aqueous layer was further extracted with 2X EtOAc. The combined organic layers were washed with water and brine and dried over anhydrous Na_2_SO_4_. Crude product was collected after solvent removal and purified.

4-benzoyl-1-*p*-chlorophenyl-3-methyl-5-pyrazol (**DA2**): Purified using FCC Hex:EtOAc 2:1, yield 61% ^1^H-NMR (CDCl_3_, δ ppm): 3.35 (s, 3H), 7.35 (d, 2H, *J* = 8.0 Hz), 7.48 (m, 5H), 8.00 (d, 2H, *J* = 8.0 Hz), ^13^C-NMR (CDCl_3_, δ ppm): 13.5, 106.1, 126.0, 127.8, 128.4, 128.8, 129.1, 133.7, 137.2, 137.6, 148.1, 160.3, 190.3 HRMS (FD+) *m*/*z*: [M]^+^ Calcd for C_17_H_13_Cl_1_N_2_O_2_ 312.0665; Found 312.0672.

Methyl,4-[[3-methyl-1-(*p*-chloroophenyl)-5-hydroxy-1H-pyrazol-4-yl]carbonyl] benzoate (**DA3**): Purified using recrystallization from EtOH/water mixture 1:4, yield 68%, ^1^H-NMR (CDCl_3_, δ ppm): 2.46 (s, 3H), 3.99 (s, 3H), 7.46 (d, 2H, *J* = 8.8 Hz), 7.59 (d, 2H, *J* = 8.8 Hz), 8.14 (d, 2H, *J* = 8.5 Hz) and 8.20 (d, 2H, *J* = 8.5 Hz), ^13^C-NMR (CDCl_3_, δ ppm): 13.6, 52.3, 106.1, 126.0, 128.8, 129.1, 129.4, 129.7, 133.7, 137.2, 137.6, 148.1, 160.3, 166.3, 190.3, HRMS (FD+) *m*/*z*: [M]+ Calcd for C_19_H_15_Cl_1_N_2_O_4_ 370.0720; Found 370.0711.

4-[[3-methyl-1-(*p*-chloroophenyl)-5-hydroxy-1H-pyrazol-4-yl]carbonyl] benzoic acid (**DA4**): For the ester hydrolysis, the procedure was adapted from literature [22]. Three molar equivalents of K_2_CO_3_ were added to the ester precursor **DA3** (1 molar equivalent) in a methanol: water (9:1) solvent mixture. The reaction was refluxed at 80 °C for 2 h. HCl (3M) was added to the cooled reaction mixture dropwise till the mixture became acidic and yellow solid started to precipitate out. The product was extracted using 3X EtOAc, washed with water and dried over anhydrous Na_2_SO_4_. Yield 88%, ^1^H-NMR (DMSO-d_6_, δ ppm): 2.26 (s, 3H), 7.51 (d, 2H, *J* = 8.8 Hz), 7.77 (d, 2H, *J* = 8.1 Hz), 7.99 (d, 2H, *J* = 8.1 Hz) and 13.15 (s, 1H broad), ^13^C-NMR (CDCl_3_, δ ppm): 14.9, 104.2, 122.7, 129.1, 130.0, 130.1, 133.0, 134.8, 136.4, 143.6, 151.7, 159.5, 167.3, 167.6, 187.2, 189.3, 197.6, 204.2, HRMS (FD+) *m*/*z*: [M]+ Calcd for C_18_H_13_Cl_1_N_2_O_4_ 356.0563; Found 356.0572.

**DA6–DA10 synthesis:**

The procedure for the synthesis of **MS208** and *O*-acyl analogues **DA6**–**DA10** was adapted from a reported procedure. The starting material **1a** (1 molar equivalent) was dissolved in anhydrous CHCl_3_. Triethylamine (1.2 molar equivalent) was added, and the stirred reaction mixture cooled down using an ice bath. The appropriate acid chloride (1.2 molar equivalent) was added dropwise while stirring. After the addition was completed, the reaction mixture was refluxed at 55 °C while stirring for 2 h. Reaction progress was monitored using TLC (EtOAc:hexane 1:2). Upon the completion of the reaction, solvent was removed under vacuum. The residue was dissolved in a small amount of CHCl_3_ and washed with water. Further aqueous layer extraction using 2X CHCl_3_ followed. Organic layers were combined and washed with water and brine and dried over anhydrous Na_2_SO_4_. CHCl_3_ was removed under vacuum to give a product which was purified using either using FCC or recrystallization using the appropriate solvent.

4-chloro-[3-methyl-1-*p*-chlorophenyl-1H-pyrazol-5-yl] benzoic acid ester (**DA6**): Purified using FCC Hex:EtOAc 1:1, yield 51%, ^1^H-NMR (CDCl_3_, δ ppm): 2.34 (s, 3H), 6.25 (s, 1H), 7.39 (d, 2H, *J* = 8.8 Hz), 7.48 (d, 2H, *J* = 8.8 Hz), 7.51 (d, 2H, *J* = 8.7 Hz), 7.99 (d, 2H, *J* = 8.7 Hz), ^13^C-NMR (CDCl_3_, δ ppm): 14.6, 96.2, 124.3, 126.2, 129.3, 129.4, 131.7, 132.9, 136.6, 141.2, 144.3, 149.5, 161.0, HRMS (FD+) *m*/*z*: [M]+ Calcd for C_17_H_12_Cl_2_N_2_O_2_ 346.0275; Found 346.0268.

[3-methyl-1-p-chlorophenyl-1H-5-pyrazol-yl-benzoate (**DA7**): Purified using FCC toluene: hexane 5:1, yield 42%, ^1^H-NMR (CDCl_3_, δ ppm): 2.35 (s, 3H), 6.27 (s, 1H), 7.39 (d, 2H, J = 8.8 Hz), 7.50 (t, 2H, J =7.5 Hz), 7.55 (d, 2H, J = 8.8 Hz), 7.67 (t, 1H, J = 7.5 Hz), 8.07 (d, 2H, J = 8.5 Hz), ^13^C-NMR (CDCl3, δ ppm): 14.6, 96.1, 124.2, 127.8, 129.0, 129.3, 130.4, 132.7, 134.5, 136.7, 149.5, 161.8, HRMS (FD+) *m*/*z*: [M]+ Calcd for C17H13Cl1N2O2 312.0665; Found 312.0666.

Methyl, 4-[[3-methyl-1-(*p*-chloroophenyl)-1H-pyrazol-5-yl]carbonyl] benzoate (**DA8**): Purified using FCC EtOAc: hexane 1:1, yield 62%, ^1^H-NMR (CDCl_3_, δ ppm): 2.46 (s, 3H), 3.99 (s, 3H), 6.39 (s, 1H), 7.46 (d, 2H, *J* = 9.0 Hz), 7.59 (d, 4H, *J* = 9.0 Hz), 8.14 (d, 2H, *J* = 8.5 Hz) and 8.19 (d, 2H, *J* = 8.5 Hz), ^13^C-NMR (CDCl_3_, δ ppm): 35.7, 52.7, 96.4, 123.6, 124.7, 129.5, 130.1, 130.4, 130.6, 131.2, 135.4, 149.4, 160.8, 165.7, HRMS (FD+) *m*/*z*: [M]+ Calcd for C_19_H_15_Cl_2_N_2_O_4_ 370.0720; Found 370.0719.

4-Chloro-[4-chloro-3-methyl-1-*p*-chlorophenyl-1H-pyrazol-5-yl] benzene sulfonic acid ester **(DA9)**: Purified using FCC EtOAc: hexane 1:1, yield 79%, ^1^H-NMR (CDCl_3_, δ ppm): 2.28 (s, 3H), 6.04 (s, 1H), 7.19 (d, 2H, *J* = 8.8 Hz), 7.29 (d, 2H, *J* = 8.5 Hz), 7.31 (d, 2H, *J* = 8.5 Hz), 7.51 (d, 2H, *J* = 8.8 Hz), ^13^C-NMR (CDCl_3_, δ ppm): 14.5, 97.7, 124.2, 129.0, 129.5, 129.7, 132.3, 133.1, 135.6, 142.1, 142.3, 149.4, HRMS (FD+) *m*/*z*: [M]+ Calcd for C_16_H_12_Cl_2_N_2_O_3_S_1_ 381.9945; Found 381.9938.

4-Sulfamoyl-[3-methyl-1-*p*-chlorophenyl-1H-pyrazol-5-yl] benzoic acid ester **(DA10)**: Purified using FCC EtOAc: hexane 1:1, yield 27%, ^1^H-NMR (DMSO-d_6_, δ ppm): 2.25 (s, 3H), 6.37 (s, 1H), 7.54 (d, 2H, *J* = 8.7 Hz), 7.66 (d, 2H, *J* = 8.7 Hz), 8.00 (d, 2H, *J* = 8.1 Hz), 8.23 (d, 2H, *J* = 8.1 Hz), ^13^C-NMR (DMSO-d_6_, δ ppm): 14.7, 97.0, 124.6, 126.4, 126.9, 129.8, 130.4, 130.5, 131.4, 131.9, 136.9, 144.4, 149.2, 149.6, 161.2, HRMS (FD+) *m*/*z*: [M]+ Calcd for C_17_H_14_Cl_1_N_3_O_4_S_1_ 391.0393; Found 391.0401.

3-[1-(4-chlorophenyl)-1H-1,2,3-triazol-4-yl]-4-chlorophenylpropanoate (**DA11**): **DA11** was synthesized following a click chemistry procedure. Crude azide, compound **3** (1 molar equivalent), and crude alkyne derivative **4** (1 molar equivalent), were dissolved in CH_2_Cl_2_. CuSO_4_·5 H_2_O (0.3 molar equivalent) and freshly prepared sodium ascorbate aqueous solution (0.3 molar equivalent) were added as single portions to the reaction mixture and left under stirring overnight at room temperature. CH_2_Cl_2_ was evaporated under vacuum. The residue was dissolved in a small amount of CH_2_Cl_2_ and washed with water. Further aqueous layer extraction using 2X CH_2_Cl_2_ followed. Organic layers were combined and washed with water and brine and dried over anhydrous Na_2_SO_4_. CH_2_Cl_2_ was removed under vacuum to give a product which purified using FCC hexane: toluene 5:1. Yield 15%, ^1^H-NMR (DMSO-d_6_, δ ppm): 3.21 (t, 2H, *J*= 6.6 Hz), 4.58 (t, 2H, *J* = 6.6 Hz), 7.60 (d, 2H, *J* = 8.6 Hz), 7.68 (d, 2H, *J* = 8.8 Hz), 7.93 (d, 2H, *J* = 8.8 Hz), 7.96 (d, 2H, *J* = 8.6 Hz), 8.77 (s, 1H), ^13^C-NMR (DMSO-d_6_, δ ppm): 25.3, 64.2, 121.6, 122.0, 129.0, 129.2, 129.4, 130.3, 131.5, 131.6, 133.2, 136.0, 138.8, 145.1, 165.3, HRMS (FD+) *m*/*z*: [M]+ Calcd for C_17_H_13_Cl_2_N_3_O_2_ 361.0384; Found 361.0389.

[1-(4-chlorophenyl)-1H-1,2,3-triazol-4-yl]-4-chlorophenylmethanol (**DA12**): **DA12** was synthesized following click chemistry procedure. Crude azide, compound **3** (1.1 molar equivalent), and crude alkyne derivative **2** (1 molar equivalent) were dissolved in t-BuOH:H_2_O 1:1 mixture. CuSO_4_·5 H_2_O (0.1 molar equivalent) and freshly prepared sodium ascorbate aqueous solution (0.3 molar equivalent) were added as single portions to the reaction mixture and left stirring overnight at room temperature. After the completion of the reaction, water was added, and product was extracted using 3X EtOAc. Organic layers were combined and washed with water and brine and dried over anhydrous Na_2_SO_4_. EtOAc was removed under vacuum to give a crude product which then purified using FCC hexane: EtOAc 5:1. Yield 65%, ^1^H-NMR (DMSO-d_6_, δ ppm): 5.90 (d, 1H, *J* = 4.5 Hz), 6.24 (d, 1H*, J* = 4.6 Hz), 7.39 (d, 2H, *J* = 8.5 Hz), 7.47 (d, 2H, *J* = 8.5 Hz), 7.63 (d, 2H, *J* = 8.9 Hz), 7.94 (d, 2H, *J* = 8.9 Hz), 8.67 (s, 1H), ^13^C-NMR (DMSO-d_6_, δ ppm): 67.6, 120.9, 122.1, 128.6, 128.8, 130.3, 132.2, 133.2, 136.0, 143.1, 153.0, HRMS (FD+) *m*/*z*: [M]+ Calcd for C_15_H_11_Cl_2_N_3_O 319.0279; Found 319.0286.

4-*p*-chlorobenzoyl-1-*p*-chlorophenyl-1H-1,2,3-triazole (**DA13**): Oxidation of **DA12** to **DA13** followed an adapted procedure from the literature [25]. One molar equivalent of **DA12** was dissolved in CH_2_Cl_2_ and 2 molar equivalents of MnO_2_ was added to the solution. The reaction mixture was stirred at room temperature overnight. The mixture was filtered over celite bed to remove MnO_2_. The filtrate was washed against water, brine and dried over anhydrous Na_2_SO_4_. The solvent was removed from the filtrate under vacuum to obtain the crude product. The residue was purified by FCC using a hexane: EtOAc solution (6:1) to give 7% of final pure compound. ^1^H-NMR (CDCl_3_, δ ppm): 7.51 (d, 2H, *J* = 8.6 Hz), 7.56 (d, 2H, *J* = 8.8 Hz), 7.76 (d, 2H, *J* = 8.8 Hz), 8.49 (d, 2H, *J* = 8.6 Hz), 8.68 (s, 1H), ^13^C-NMR (CDCl_3_, δ ppm): 122.0, 126.4, 128.9, 130.3, 132.2, 134.5, 135.6, 140.2, 184.0, HRMS (FD+) *m*/*z*: [M]+ Calcd for C_15_H_9_Cl_2_N_3_O 317.0122; Found 317.0116.

### 3.2. Expression and Purification of MtbUGM

A vector construct [pET-29b (+)- His_6_], containing the gene encoding for *Mtb*UGM (provided by Prof. Laura L. Kiessling; Massachusetts Institute of Technology), was transformed into GOLD BL21 *E. coli* cells [32]. Transformed cells were grown in Terrific Broth with 50 μg/mL kanamycin at 37 °C and culture overnight without induction. The cells were harvested by centrifugation at 3500 rpm for 30 min at 4 °C. The pellet was resuspended in lysis buffer (20 mM sodium phosphate, 500 mM sodium chloride, pH 8.0). Cell lysis was achieved by incubating the suspended pellet with lysozyme (20 µg/mL), 1% Triton-X 100, DNase (10 µg/mL) and AEBSF (20 µg/mL) for 30 min at 4 °C followed by sonication (40%, 15 s on, 15 s off, 4 min total time). Lysed cells were centrifuged at 15,000 rpm for 45 min at 4 °C. Purification of the soluble fraction was completed using nickel affinity chromatography. The soluble fraction was loaded onto a nickle affinity column, His GraviTrap column (GE-Healthcare). The column was washed with a 50 mM phosphate buffer containing 300 mM NaCl pH 8.0 followed by a second wash of 50 mM phosphate buffer containing 300 mM NaCl pH 8.0 and 50 mM imidazole, pH 8.0. The protein was eluted using 50 mM sodium phosphate buffer pH 8.0 containing 300 mM NaCl and 250 mM imidazole. Fractions containing the pure protein were collected and dialyzed overnight against 20 mM sodium phosphate buffer containing 150 mM sodium chloride pH 8.0 at 4 °C. The purity of the protein was estimated by SDS-PAGE and the concentration was measured using the NanoDrop (Nanodrop Technologies, ND-1000 UV/Vis Spectrophotometer, Delaware, USA) at 230 nm. A 1 L bacterial culture produced around 20 mg pure protein [33].

### 3.3. Inhibition Assays

For the evaluation of the kinetic inhibition activities of the target compounds, a reported discontinuous assay was followed [34]. The conversion of UDP-Gal*f* to UDP-Gal*p* was monitored using High Performance Liquid Chromatography (HPLC) in a 100 µL total reaction volume.

UDP-Gal*f* was synthesized and generously provided by Dr. Todd Lowary (University of Alberta). The concentration of UDP-Gal*f* stock solution was calibrated against standard UDP-Gal*p* using HPLC. The reaction vials were initially degassed with argon. Carbopac PA1 (Dionix Inc) ion exchange column was pre-equilibrated with 200 mM ammonium acetate pH 7.0 buffer in Agilent 1100 Infinity HPLC system. Both substrate and product were detected at 262 nm.

#### 3.3.1. Determination of % Inhibition

*Mtb*UGM (final concentration 10 nM), was incubated for 2 min at 37 °C in 500 mM sodium phosphate buffer pH 7.0. Reduction of the cofactor FAD was achieved by using freshly prepared 20 mM sodium dithionite to the reaction mixture. After 30 s at 37 °C, 60 µM final concentration of the test compound in DMSO was added, where DMSO was 6% *v*/*v* in the final reaction mixture. The reaction was left for an additional minute at 37 °C before the addition of 15 μM final concentration of UDP-Gal*f*. The reaction was left at 37 °C to proceed for the appropriate amount of time needed to give a 30–40% conversion. The reaction was then quenched with 100 µL of n-butanol. The aqueous layer was collected and analyzed by HPLC using 200 mM ammonium acetate pH 7.0 as the isocratic elution buffer. The results were controlled against the presence of DMSO. All of the experiments were conducted in duplicate. The corresponding rate and the % inhibition were calculated from the Equations (1)–(3):

(1) $$\%\;conversion=\frac{AUC\;UDP\_Galp\;peak}{AUC\;UDP\_Galf\;peak+AUC\;UDP\_Galp\;peak}\times 100$$

(2) $$Initial\;velocity=\frac{\%\;\mathrm{conversion}\;\times \;\mathrm{substrate}\;\mathrm{concentration}\;\left(\mu M\right)}{\;\mathrm{time}\;\left(\sec\right)}$$

(3) $$\%\;inhibition=\frac{Initial\;velocity\;with\;the\;inhibitor-Initial\;velocity\;without\;the\;inhibitor\;}{Initial\;velocity\;without\;the\;inhibitor}\times 100\;$$

#### 3.3.2. Determination of Inhibition Model

The previously mentioned procedure was repeated but with varying UDP-Gal*f* concentrations (10, 20, 40, 80, 100, and 150 μM) and changing **DA10** concentrations (200 and 400 μM). Uninhibited *Mtb*UGM activity in presence of 6% *v*/*v* DMSO was used as the negative control experiment. The corresponding reaction rate at each substrate and inhibitor concentration was calculated using Equations (1) and (2).

Collected data of reaction rates were plotted against UDP-Gal*f* concentrations for each inhibitor concentration. Kinetic parameters, including V_max_ and *K*_M_ from non-linear regression fitting, were calculated using the SigmaPlot software (SigmaPlot 12.0). The type of inhibition of the test compound was analyzed using SigmaPlot and the Dixon and Cornish-Bowden Plots were created using Microsoft Excel.

### 3.4. Antituberculosis Activity Determination

To a 96-well microplate, 98 µL of Middlebrook 7H9 broth was added to wells B2-G2 while 50 µL was added to the remaining wells B3-G11. 200 µL of sterile ddH_2_O was added to the perimeter wells to minimize evaporation of medium in test wells during incubation and mitigate edge effects on *M. tuberculosis* growth. 2 µL of 10 mg/mL working solutions of test compounds were added to wells B2-F2 to give starting concentrations of 200 µg/mL. The working solution (2 µL of 200 µg/mL) of the positive control isoniazid INH (Sigma I3377-5G, St. Louis, MO, USA) (in ddH_2_O) was added to well G2 to give a starting concentration of 4200 µg/mL. 50 µL starting from wells B2-G2 was transferred serially to wells B10-G10 for range of nine 2-fold dilutions. The excess 50 µL from wells B10-G10 was discarded so that each well has 50 µL remaining. Wells B11-G11 without test compounds or INH served as no treatment controls. An inoculation mixture containing 6 × 10^4^ CFU/mL of *Mtb* in 7H9 broth was prepared. 50 µL of this inoculation mixture was added to wells B2-G11 so that each well received 3 × 10^3^ CFU/mL of *M. tuberculosis.* Final concentration range of test compounds were: 200, 100, 50, 25, 12.5, 6.25, 3.125, 1.5625, and 0.78125 µg/mL; INH: 4, 2, 0.5, 0.25, 0.125, 0.0625, 0.03125, and 0.015625 µg/mL. The microplates were lidded, placed in zip-loc bags, and incubated at 37 °C for 7 days (~10 doublings).

After 7 days, 10 µL of resazurin (Sigma R7017-5G, St. Louis, MO, USA) (at 0.025% *w*/*v* in ddH_2_O and filter-sterilized) was added to wells B2-G11 and incubated at 37^o^C overnight. Growth of *M. tuberculosis* in the plates, revealed by conversion of resazurin to resorufin, was assessed visually and by fluorescence measurement in a microplate reader (Molecular Devices SpectraMax i3x). Two independent REMAs for each test sample were performed.

## 4. Conclusions

Seven of the synthesized compounds inhibited the growth of *M. tuberculosis* in vitro. However, none of the introduced structural modifications to **MS208** improved the antituberculosis activity. All of the compounds showed inhibition against *Mtb*UGM except for **DA6** and **DA7**. **DA10** was a promising *Mtb*UGM inhibitor which was shown to be a competitive inhibitor and not a mixed inhibitor such as the lead compound **MS208**. Further studies are needed to understand more about the difference in the inhibition mechanism and to better establish the SAR.

## Figures and Tables

**Table 1:** MICs of the synthesized compounds as determined by REMA. Isoniazid (INH) was used a positive control.

Test Compound	MIC (µg/mL)
**MS208**	12.5
**DA1**	12.5
**DA2**	12.5
**DA3**	25
**DA4**	>100
**DA5**	25
**DA6**	50
**DA7**	>100
**DA8**	50
**DA9**	>100
**DA10**	>100
**DA11**	>100
**DA12**	50
**DA13**	>100
**INH**	0.25

## Data Availability

Data is contained within the article or Supplementary Materials.

## Supplementary Materials

The ^1^H&^13^C-NMR spectra of **DA4**–**DA13** are available online at www.mdpi.com/article/10.3390/ph15020197/s1.
