# Supplementary material for: Pyrazole and Triazole Derivatives as Mycobacterium tuberculosis UDP-Galactopyranose Inhibitors
Source: Pharmaceuticals (Basel). 2022 Feb 4;15(2):197. doi: 10.3390/ph15020197 (PMC8874540; doi:10.3390/ph15020197)

Supplementary material

Pyrazole and Triazole derivatives as Mycobacterium tuberculosis UDP-galactopyranose inhibitors

Dalia M. Ahmed[1,2], Jeffrey M. Chen[3,4,5],David A. R. Sanders[1*]

[1] Department of Chemistry, University of Saskatchewan, 110 Science Place, Saskatoon, Saskatchewan, Canada S7N 5C9, dalia.ahmed@usask.ca

[2] Pharmaceutical Chemistry Department, Faculty of Pharmacy, Ain Shams University, Egypt

[3] Vaccine and Infectious Disease Organization, Saskatoon, Saskatchewan S7N 5E3, Canada, jeffrey.chen@usask.ca

[4] Department of Veterinary Microbiology, Western College of Veterinary Medicine, University of Saskatchewan, Saskatoon, Saskatchewan S7N 5B4, Canada.

[5] Vaccinology and Immunotherapeutics Program, School of Public Health, University of Saskatchewan, Saskatoon, Saskatchewan S7N 2Z4, Canada.

* Correspondence: david.sanders@usask.ca

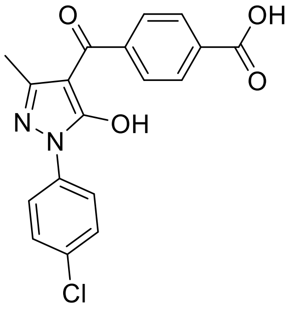
O
OH
O
N
N
OH
Cl

DA4

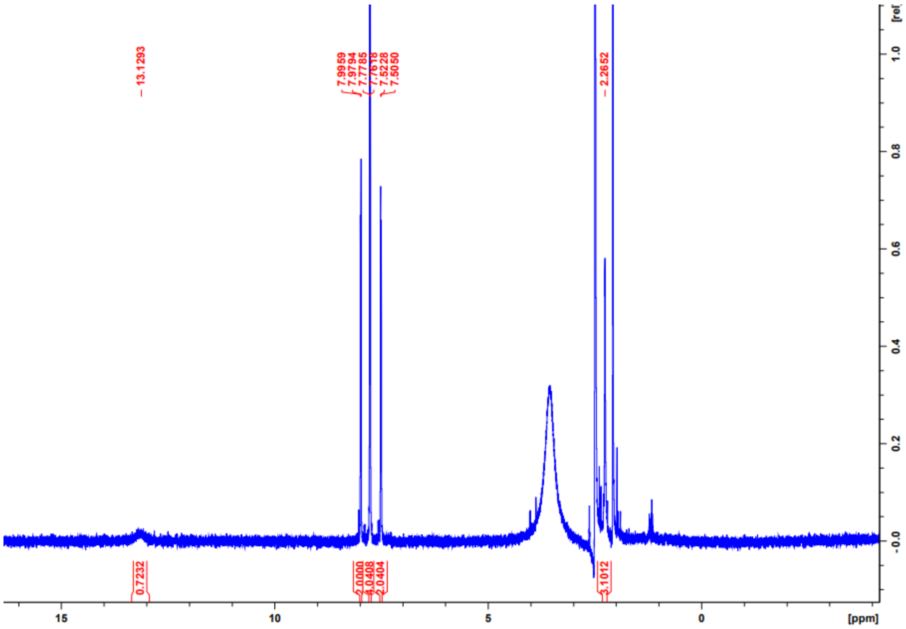
13.1293
7.9959
7.9794
7.7785
7.5228
7.5050
2.2652
0.7232
2.0000
4.0408
2.0404
3.1012
[ppm]
[rel]

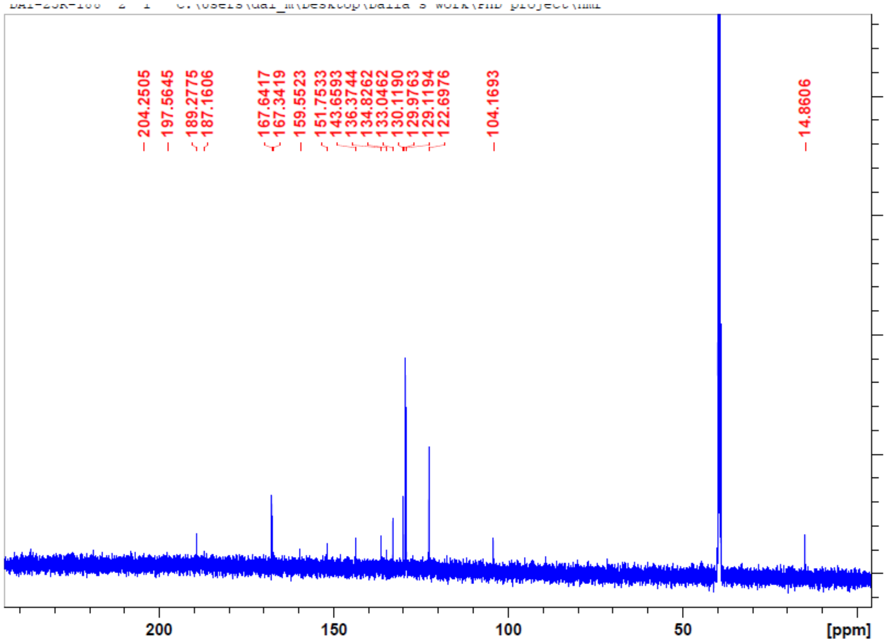
204.2505
197.5645
189.2775
187.1606
167.6417
167.3419
159.5523
151.7533
143.6593
136.3744
134.8262
133.0462
130.1190
129.9763
129.1194
122.6976
104.1693
14.8606
[ppm]
[rel]

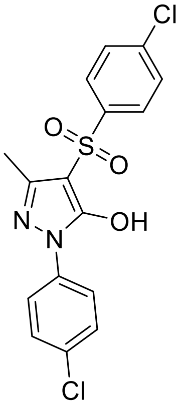
Cl
O
S
O
N
N
OH
Cl

DA5

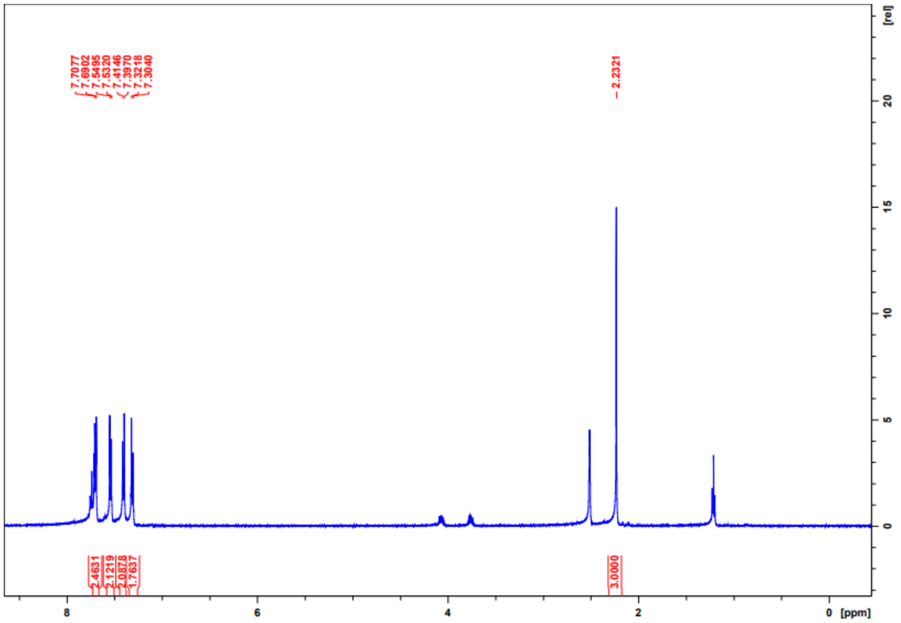
7.7077
7.6902
7.5495
7.5320
7.4146
7.3970
7.3218
7.3040
2.2321
2.4631
2.1219
2.0878
1.7637
3.0000
[rel]
[ppm]

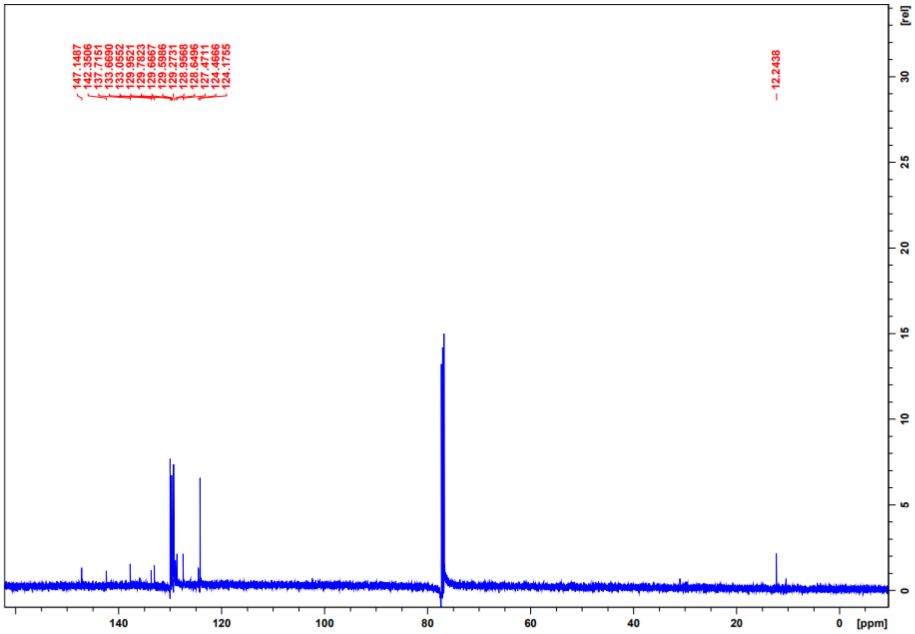
147.1487
142.3506
137.7151
133.6690
133.0552
129.9521
129.7823
129.6667
129.5986
129.2731
128.9568
128.6496
127.4711
124.4666
124.1755
12.2438
[rel]
[ppm]

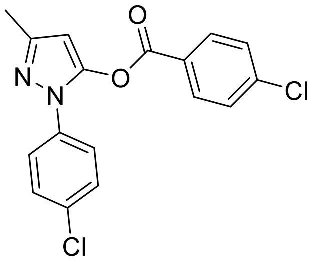
O
N
N
O
Cl
Cl

DA6

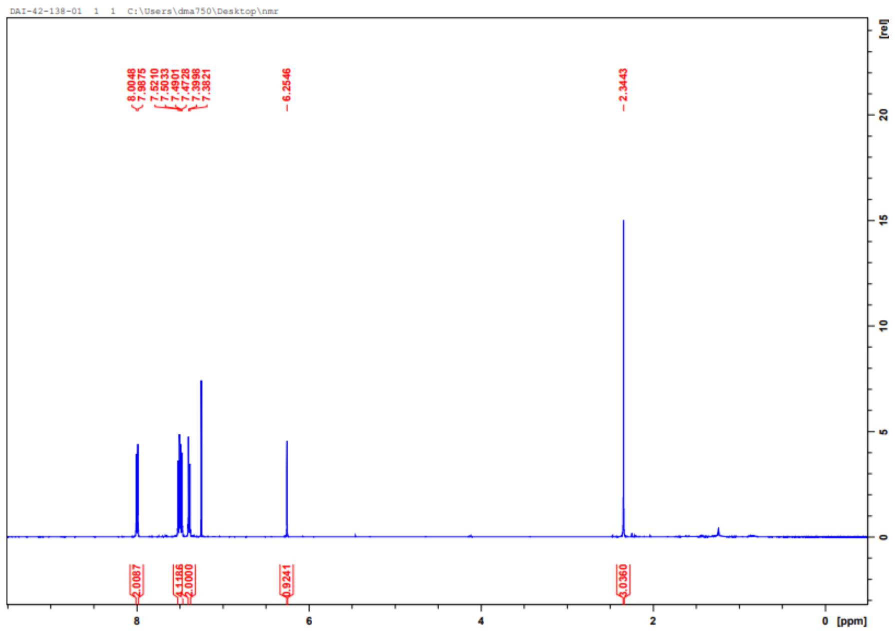
DAI-42-138-01 1 1 C:\Users\dma750\Desktop\nmr
8.0048
7.9875
7.5210
7.5033
7.4901
7.4728
7.3998
7.3821
6.2546
2.3443
2.0087
4.1166
2.0000
0.9241
3.0360
[rel]
[ppm]

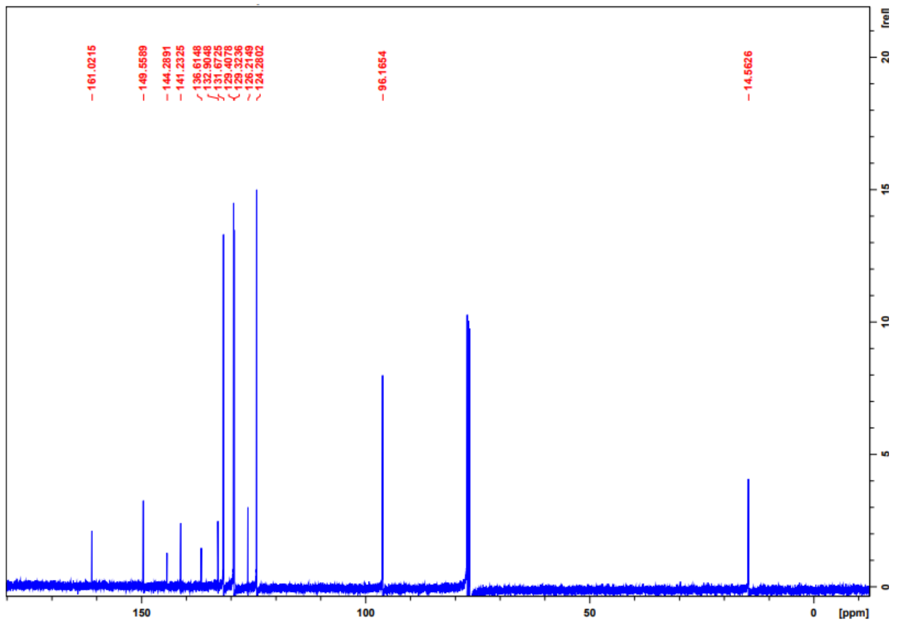
161.0215
149.5589
144.2891
141.2325
136.6148
132.9048
131.6725
129.4078
129.3236
126.2149
124.2802
96.1654
14.5626
[rel]
[ppm]

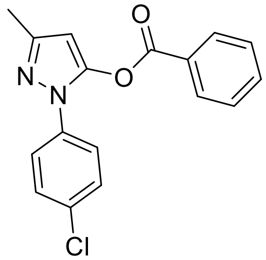
O
N
N
O
Cl

DA7

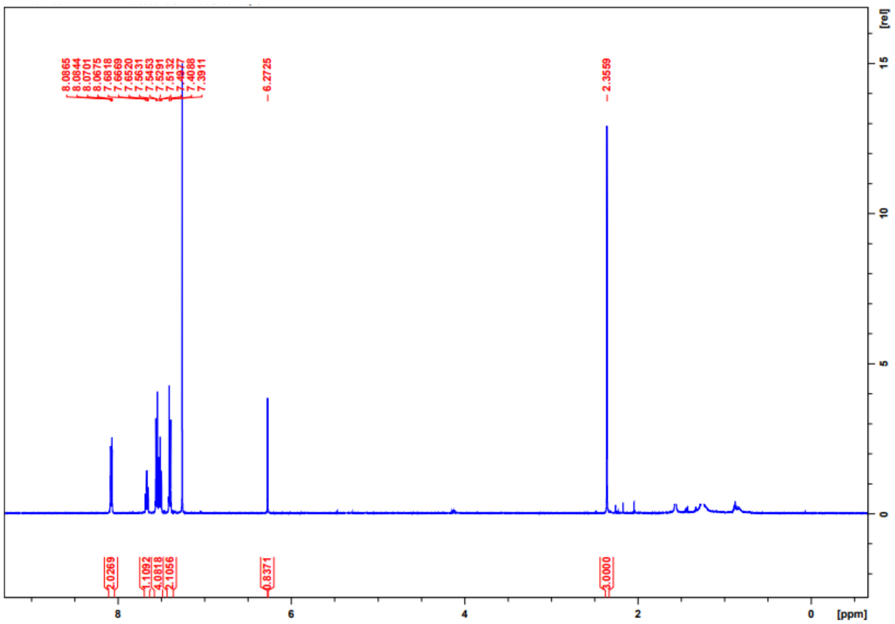
8.0865
8.0844
8.0701
8.0675
7.6818
7.6669
7.6520
7.5631
7.5453
7.5291
7.5132
7.4927
7.4088
7.3911
6.2725
2.3559
2.0269
1.1092
4.0818
2.1056
0.8371
3.0000
[rel]
[ppm]

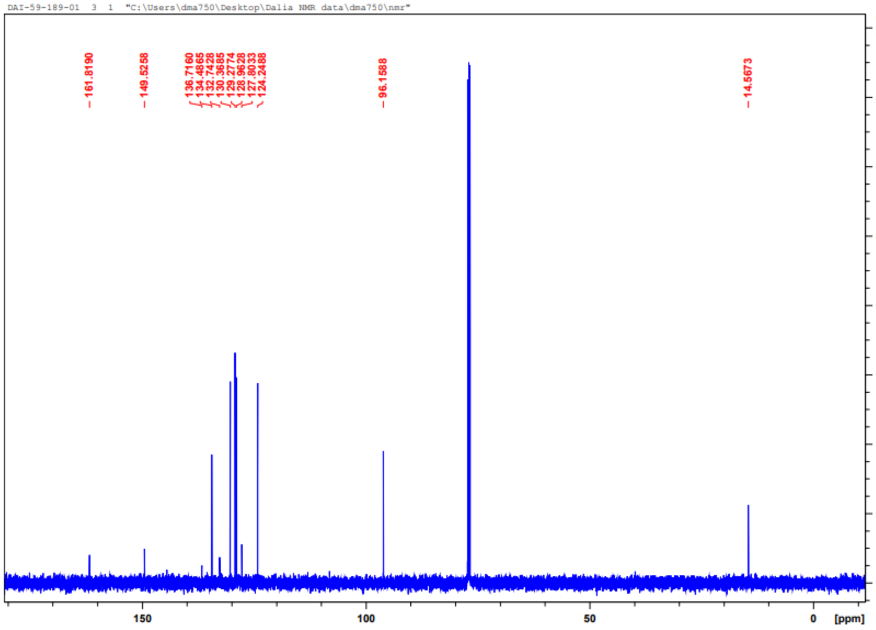
DAI-59-189-01 3 1 "C:\Users\dma750\Desktop\Dalia NMR data\dma750\nmr"
161.8190
149.5258
136.7160
134.4865
132.7428
130.3685
129.2774
128.9628
127.8033
124.2488
96.1588
14.5673
[rel]
[ppm]

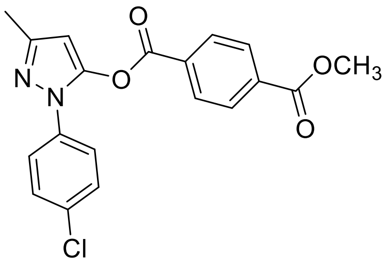
O
O
OCH3
N
N
O
Cl

DA8

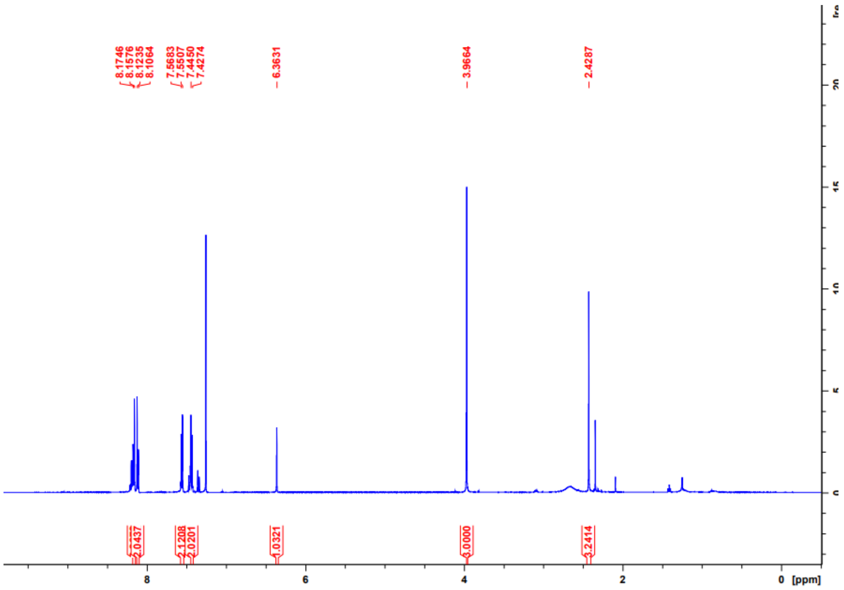
8.1746
8.1576
8.1235
8.1064
7.5683
7.5507
7.4450
7.4274
6.3631
3.9664
2.4287
2.0437
2.1208
2.0201
1.0321
3.0000
3.2414
8
6
4
2
0 [ppm]

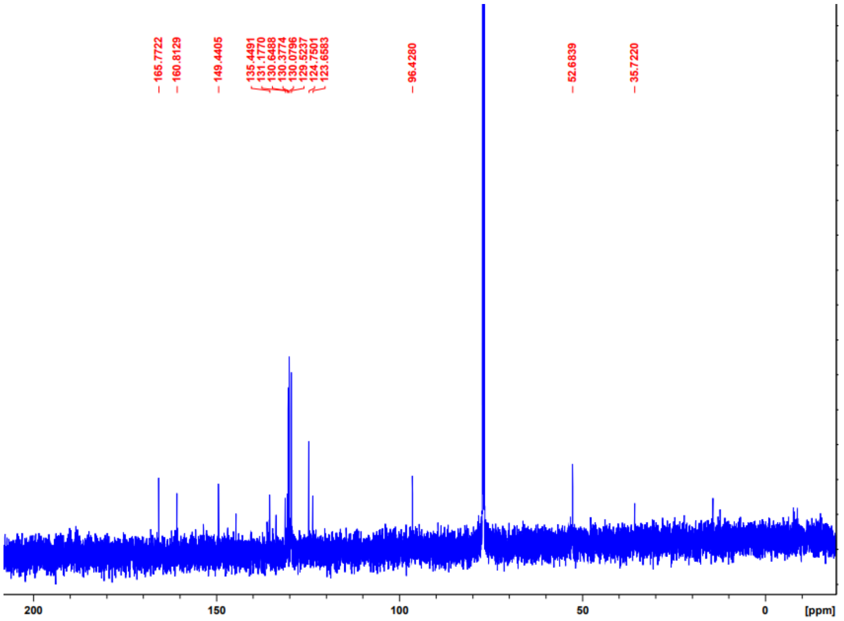
165.7722
160.8129
149.4405
135.4491
131.1770
130.6488
130.3774
130.0796
129.5237
124.7501
123.6583
96.4280
52.6839
35.7220
200
150
100
50
0
[ppm]

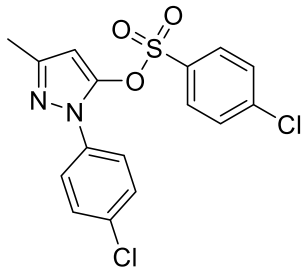
O
O
S
O
N
N
Cl
Cl

DA9

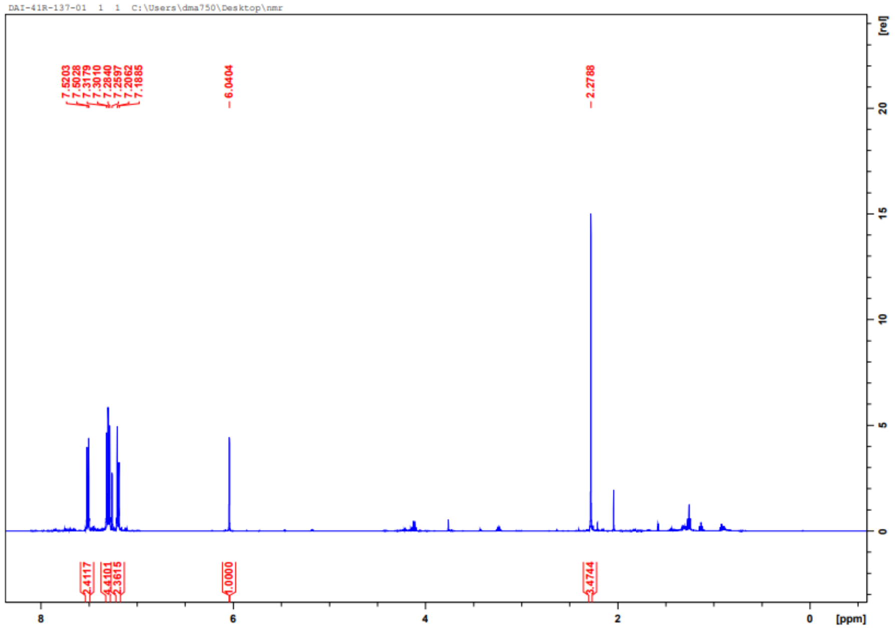
DAI-41R-137-01 1 1 C:\Users\dma750\Desktop\nmr
7.5203
7.5028
7.3179
7.3010
7.2840
7.2597
7.2062
7.1885
6.0404
2.2788
2.4117
4.4101
2.3615
1.0000
3.4744
[ppm]
[rel]

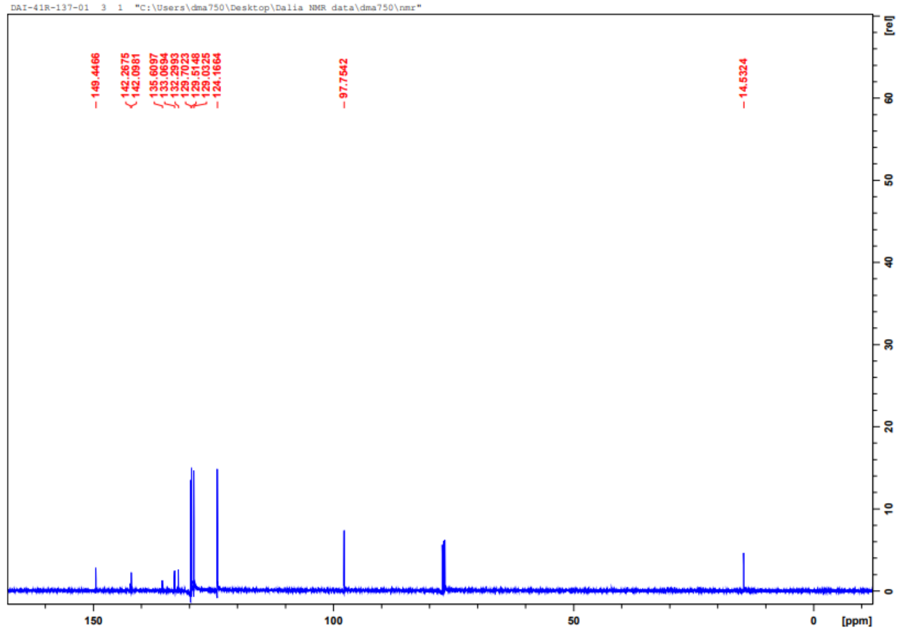
DAI-41R-137-01 3 1 "C:\Users\dma750\Desktop\Dalia NMR data\dma750\nmr"
149.4466
142.2675
142.0981
135.6097
133.0694
132.2993
129.7023
129.5148
129.0326
124.1664
97.7542
14.5324
[ppm]
[rel]

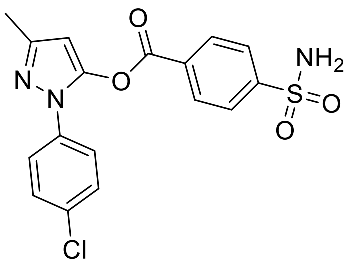
O
NH2
N
N
O
S
O
O
Cl

DA10

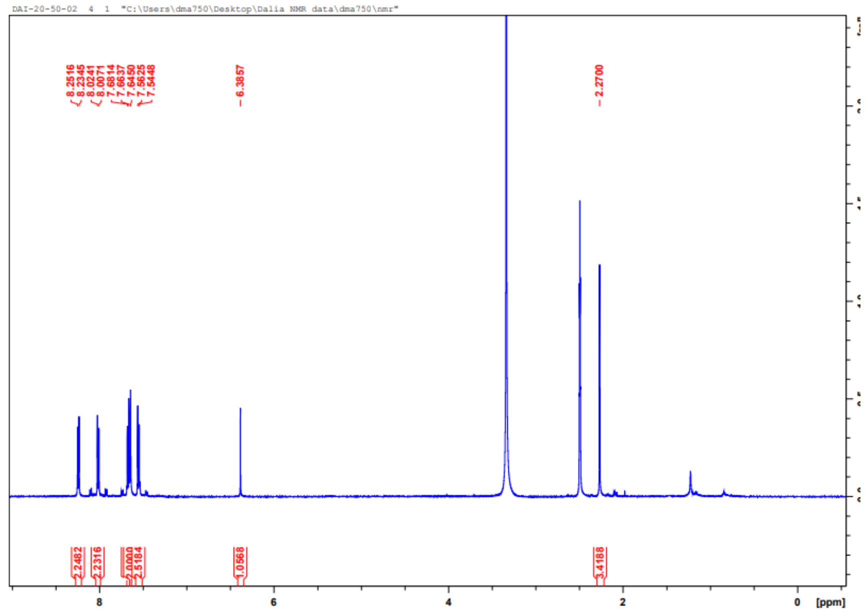
DAI-20-50-02 4 1 "C:\Users\dma750\Desktop\Dalia NMR data\dma750\nmr"
8.2516
8.2345
8.0241
8.0071
7.6814
7.6637
7.6450
7.5625
7.5448
6.3857
2.2700
2.2482
2.2316
2.0000
2.5184
1.0568
3.4188
8
6
4
2
0
[ppm]

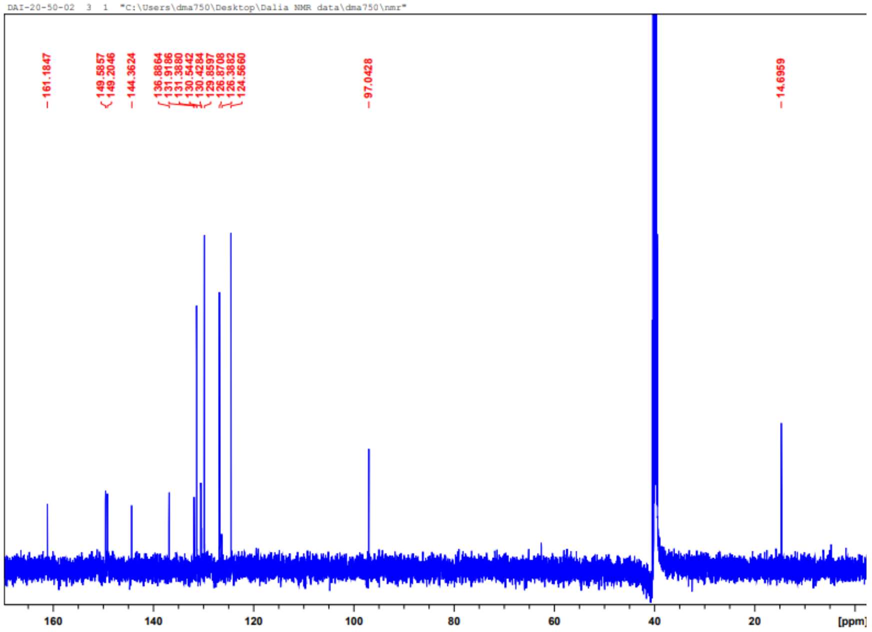
DAI-20-50-02 3 1 "C:\Users\dma750\Desktop\Dalia NMR data\dma750\nmr"
161.1847
149.5857
149.2046
144.3624
136.8864
131.9186
131.3880
130.5442
130.4284
129.8597
126.8708
126.3882
124.5660
97.0428
14.6959
160
140
120
100
80
60
40
20
[ppm]

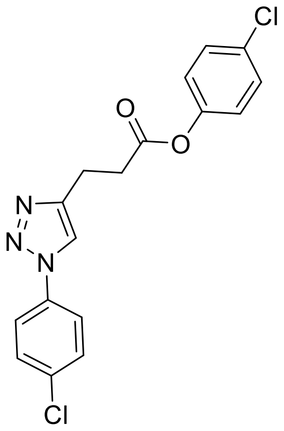
Cl
O
O
N
N
N
Cl

DA11

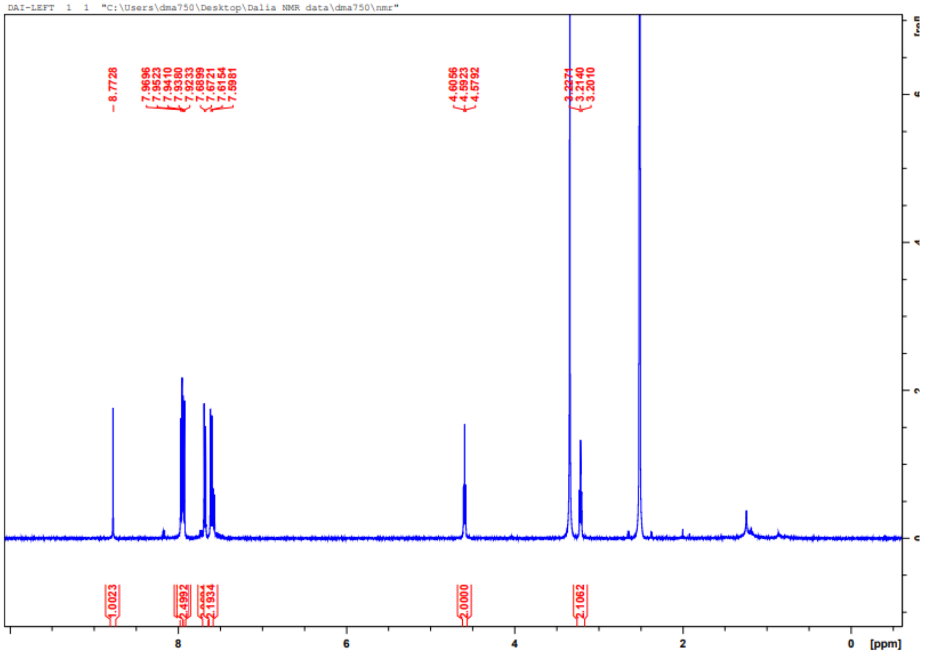
DAI-LEFT 1 1 "C:\Users\dma750\Desktop\Dalia NMR data\dma750\nmr"
8.7728
7.9696
7.9523
7.9410
7.9380
7.9233
7.6899
7.6721
7.6154
7.5981
4.6056
4.5923
4.5792
3.2271
3.2140
3.2010
1.0023
2.4992
2.1934
2.0000
2.1062
8
6
4
2
0
[ppm]

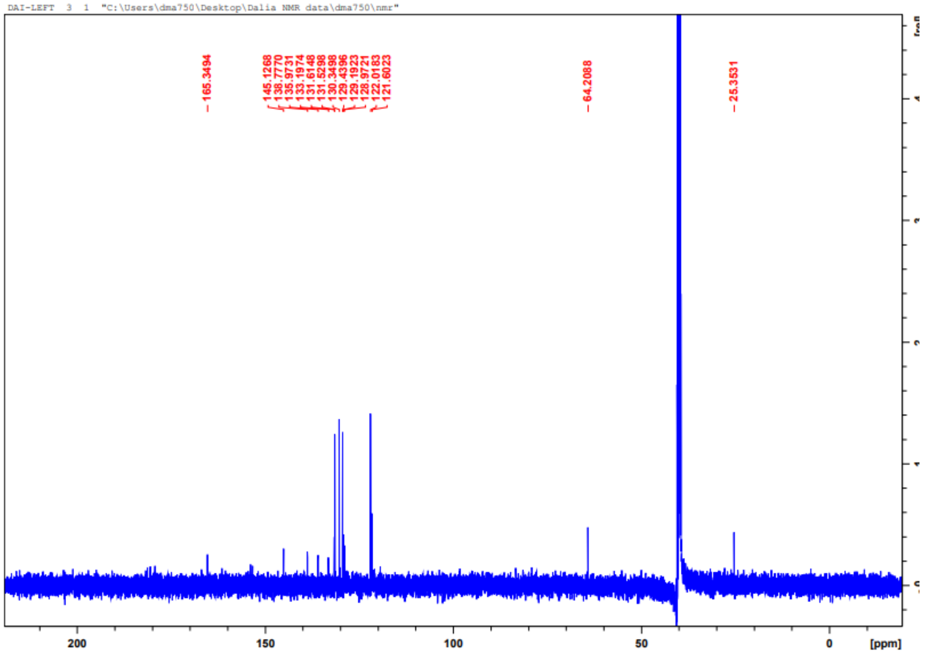
DAI-LEFT 3 1 "C:\Users\dma750\Desktop\Dalia NMR data\dma750\nmr"
165.3494
145.1268
138.7770
135.9731
133.1974
131.6148
131.5298
130.3498
129.4396
129.1923
128.9721
122.0183
121.6023
64.2088
25.3531
200
150
100
50
0
[ppm]

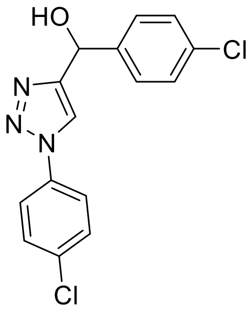
HO
Cl
N
N
N
Cl

DA12

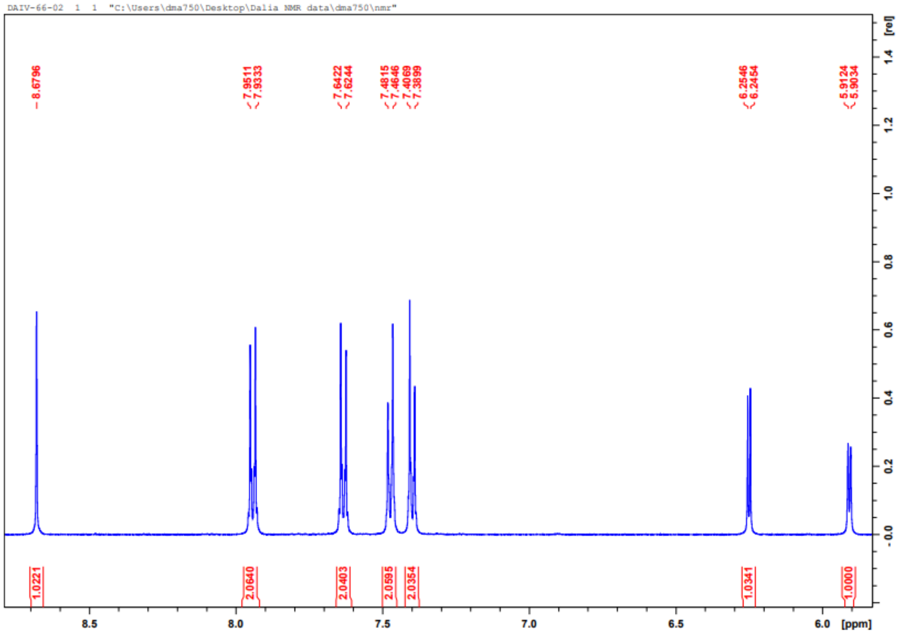
DAIV-66-02 1 1 "C:\Users\dma750\Desktop\Dalia NMR data\dma750\nmr"
8.6796
7.9511
7.9333
7.6422
7.6244
7.4815
7.4646
7.4069
7.3899
6.2546
6.2454
5.9124
5.9034
1.0221
2.0640
2.0403
2.0595
2.0354
1.0341
1.0000
[ppm]
[rel]

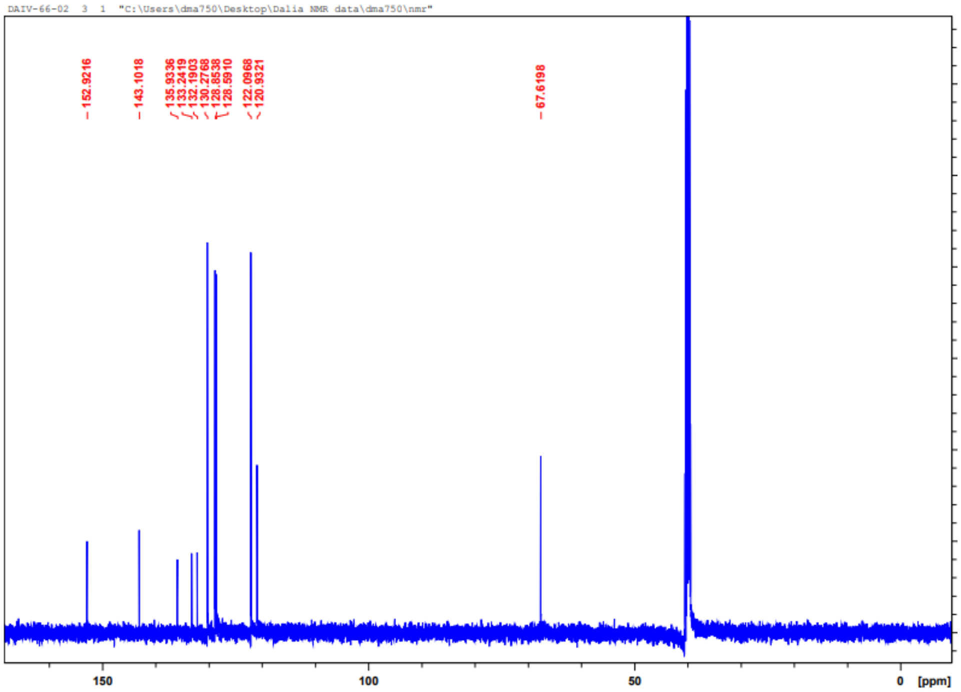
DAIV-66-02 3 1 "C:\Users\dma750\Desktop\Dalia NMR data\dma750\nmr"
152.9216
143.1018
135.9336
133.2419
132.1903
130.2768
128.8538
128.5910
122.0968
120.9321
67.6198
[ppm]

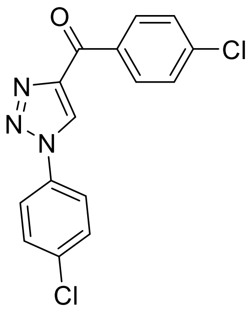

DA13

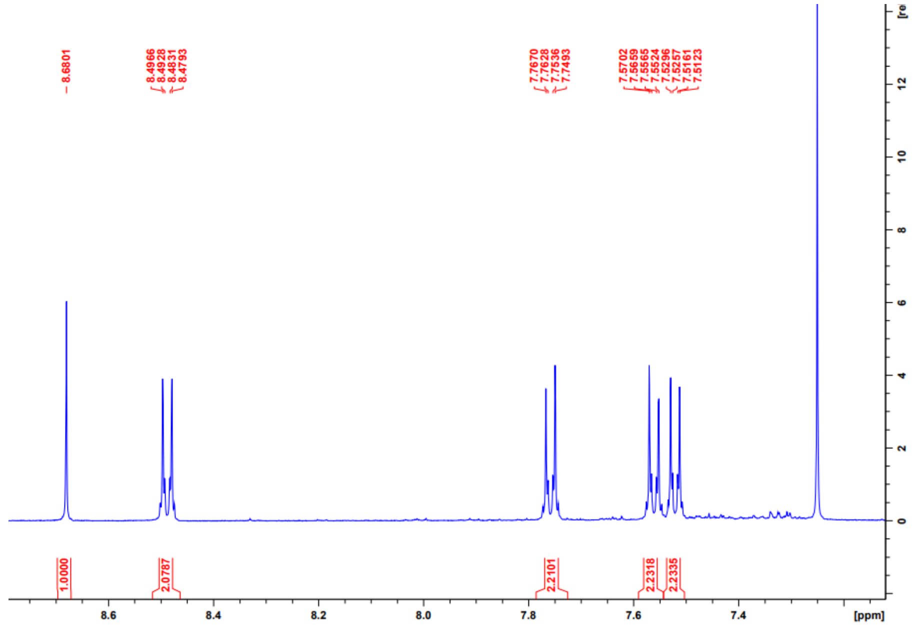

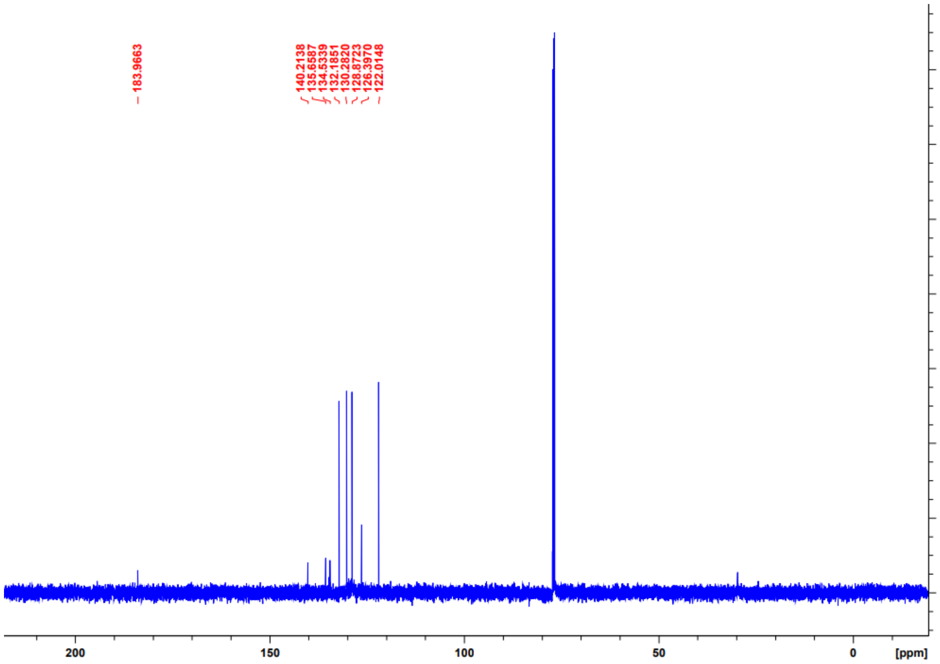

Supplement: Supplementary file 1 [file pharmaceuticals-15-00197-s001.zip › pharmaceuticals-1582235-supplementary.pdf]
